# A phase II study of FOLFOX combined with nab-paclitaxel in the treatment of metastatic or advanced unresectable gastric, gastroesophageal junction adenocarcinoma: a Big Ten Cancer Research Consortium trial

**DOI:** 10.1093/oncolo/oyae236

**Published:** 2024-09-18

**Authors:** Marie S Dreyer, Mary Mulcahy, Masha Kocherginsky, Yolande Chen, Howard S Hochster, Pashtoon M Kasi, Sheetal Kircher, Emil Lou, Yangruijue Ma, Nataliya V Uboha, Al B Benson

**Affiliations:** Robert H. Lurie Comprehensive Cancer Center, Northwestern University, Chicago, IL, United States; Robert H. Lurie Comprehensive Cancer Center, Northwestern University, Chicago, IL, United States; Division of Biostatistics, Department of Preventive Medicine, Feinberg School of Medicine, Northwestern University, Chicago, IL, United States; University of Illinois at Chicago, Chicago, IL, United States; Rutgers Cancer Institute of New Jersey, New Brunswick, NJ, United States; University of Iowa, Iowa City, IA, United States; Robert H. Lurie Comprehensive Cancer Center, Northwestern University, Chicago, IL, United States; University of Minnesota School of Medicine, Minneapolis, MN, United States; Division of Biostatistics, Department of Preventive Medicine, Feinberg School of Medicine, Northwestern University, Chicago, IL, United States; University of Wisconsin Carbone Cancer Center, Madison, WI, United States; Robert H. Lurie Comprehensive Cancer Center, Northwestern University, Chicago, IL, United States

**Keywords:** gastroesophageal, gastric, adenocarcinoma, nab-paclitaxel, fluorouracil, oxaliplatin, metastatic, HER2-negative

## Abstract

**Background:**

Doublet platinum or taxane-based therapies are the current standard backbone of treatment for advanced gastric/gastroesophageal junction (GEJ) adenocarcinoma. Previously used anthracycline-based triplet regimens are no longer used routinely due to toxicity and lack of superior efficacy. We hypothesized that the addition of nab-paclitaxel to FOLFOX (FOLFOX-A) would induce higher efficacy and better tolerability.

**Patients and Methods:**

Eligible patients with chemotherapy-naïve advanced unresectable HER2-negative gastric or GEJ adenocarcinoma were enrolled in this phase II single-arm trial of FOLFOX (oxaliplatin 85 mg/m^2^, leucovorin 400 mg/m^2^, 5-FU 2400 mg/m^2^ over 46-48 hours) + nab-paclitaxel (150 mg/m^2^) every 14 days of a 28-day cycle. Evaluable disease according to RECIST v1.1 for solid tumors was required. The primary endpoint was the objective response rate. Simon’s optimal 2-stage design was used to test 5% versus 20% response rate with 90% power and 10% one-sided type I error rate.

**Results:**

The study enrolled 39 patients. Median age was 63 (range 20-80) years, 30 (77%) were male, 34 (94%) were White, and 21 (57%) had gastric tumors. The median number of cycles completed was 4.5 (range: 0-36), and 25 patients required dose reductions or discontinuation of at least one component due to toxicity. Of the 38 patients evaluable for response, 15 (42.9%) had complete/partial response (CR/PR) as the best response, and 13 (37.1%) had stable disease. progression-free survival (PFS) and OS data were available for 38 patients, with a median follow-up duration of 27 months (range: 18.2-51.9 months for censored patients). Median PFS was 6.6 months (95% CI: 5.6-12.9), with 31.0% (95% CI: 18.4%-52.4%) 12-month PFS rate. The median OS was 10.5 months (95% CI: 8.8-20.7), 12-month OS rate was 44.7% (95% CI: 31.4%-63.7%). Treatment-related grade 3-4 toxicities included peripheral sensory neuropathy and anemia (18.4% each), neutropenia (15.8%), and diarrhea and lymphopenia (7.9% each).

**Conclusions:**

FOLFOX-A has a significant response rate, expected toxicities, and should be considered for future investigation in combination with immunotherapy given the recent approvals.

## Teaser

This phase II trial evaluated the efficacy and safety of the combination of FOLFOX and nab-paclitaxel as first-line therapy for patients diagnosed with advanced HER2-negative gastric or gastroesophageal junction adenocarcinoma.

**Implications for Practice**

This open-label, single-arm phase II multi-institutional trial evaluated the efficacy and safety of the combination of FOLFOX and nab-paclitaxel (FOLFOX-A) as first-line therapy for patients diagnosed with advanced HER2-negative gastric or gastroesophageal junction (GEJ) adenocarcinoma. FOLFOX-A showed an overall objective response of 42.9% exceeding the expected study target of 20%, had expected toxicities, and extended disease control for some patients. FOLFOX-A demonstrated a more favorable hematologic toxicity profile than historical controls for other triplet regimens. Future investigations could consider incorporating FOLFOX-A chemotherapy backbone in combination with immunotherapy.

## Introduction

Gastric cancer is the fifth most prevalent malignancy worldwide leading to the third and fifth leading cause of cancer death in men and women, respectively.^1^ A steady decline in stomach cancer incidence and mortality rates has been observed in most developed countries of Northern America and Europe since the mid-20th century. However, adenocarcinoma of the gastric cardia is increasing in Northern America and Europe, perhaps due to increased obesity and/or improvements in classification.

Clinical outcomes for gastric cancer remain poor, with a 5-year survival rate of 35% across all stages. Most patients are diagnosed with advanced disease, making surgical resection either not feasible or available with purely palliative intent. Combination chemotherapy is the mainstay of therapy with a median survival typically around 1 year compared with 3-4 months for those treated with supportive care alone.^2^

Doublet chemotherapy with fluoropyrimidine (fluorouracil or capecitabine) combined with either oxaliplatin or cisplatin, such as FOLFOX, are preferred regimens due to their efficacy and tolerability.^3-7^ Triplet regimens with taxane- or epirubicin-based therapy have shown efficacy in patients with locally advanced or metastatic gastric cancer, but the less favorable toxicity profile is prohibitive.^8,9^ Recent advances have identified a benefit in HER2, PD-L1, and mismatch repair-directed therapy added to doublet chemotherapy for specific cases.

Nab-paclitaxel, a solvent-free, albumin-bound colloidal formulation of paclitaxel, offers a potential solution for achieving more effective and more tolerable triplet therapy. Nab-paclitaxel has shown antitumor activity in other advanced cancer types and has received FDA approval for use in breast,^10^ non–small cell lung,^11^ and pancreatic cancers.^12^ Additionally, the combination of FOLFOX and nab-paclitaxel (FOLFOX-A) has been well-studied in advanced pancreatic adenocarcinoma.^13-15^

Strategies to improve response, survival, and quality of life for patients with advanced gastric cancer not harboring actionable genetic or other targetable alterations are needed. To fill this important clinical need, we evaluated FOLFOX-A in metastatic or advanced unresectable HER2-negative gastric and GEJ adenocarcinoma. We hypothesized that this combination (FOLFOX-A) would have a higher efficacy and better tolerability.

## Patients and methods

### Study design and patients

This is an open-label, single-arm phase II multi-institutional trial designed to evaluate the efficacy and safety of the FOLFOX-A as first-line therapy for patients diagnosed with histologically confirmed advanced HER2-negative gastric or gastroesophageal junction (GEJ) adenocarcinoma.

Eligible patients were age 18 years or older with pathologically documented advanced, metastatic, or unresectable gastric/GEJ adenocarcinoma; had no previous treatment for metastatic disease (allowed to have prior neoadjuvant or adjuvant treatment completed greater than 6 months prior to metastatic disease), adequate bone marrow and organ function; and had Eastern Cooperate Oncology Group performance status (ECOG PS) scores of 0 or 1. Patients were excluded from the study if they had HER2-positive tumors; preexisting peripheral neuropathy; active infection; known hypersensitivity to fluorouracil (5-FU), oxaliplatin, or other platinum agents; known hypersensitivity to nab-paclitaxel or any of its excipients; significant pulmonary conditions; or uncontrolled intercurrent illness. Patients with known active central nervous system metastases and/or carcinomatous meningitis were also excluded; however, patients with stable previously treated brain metastases were allowed to participate.

All patients received FOLFOX-A on days 1 and 15 of each cycle (1 cycle = 28 days). Nab-paclitaxel was given at a dose of 150 mg/m^2^ intravenous (IV) over 30-40 minutes, followed by oxaliplatin 85 mg/m^2^ IV over 120 minutes, leucovorin 400 mg/m^2^ IV over 2 hours, and 5-FU as a continuous IV infusion over day 1 and day 2 (for a total dose of 2400 mg/m^2^ over 46-48 hours). Patients were allowed to receive premedication, including dexamethasone 20 mg within 12 hours prior to the nab-paclitaxel and/or diphenhydramine (25-50 mg IV) given 30 minutes prior to nab-paclitaxel or per institutional standards. Patients continued treatment until disease progression or unacceptable toxicity.

**Figure 1.**
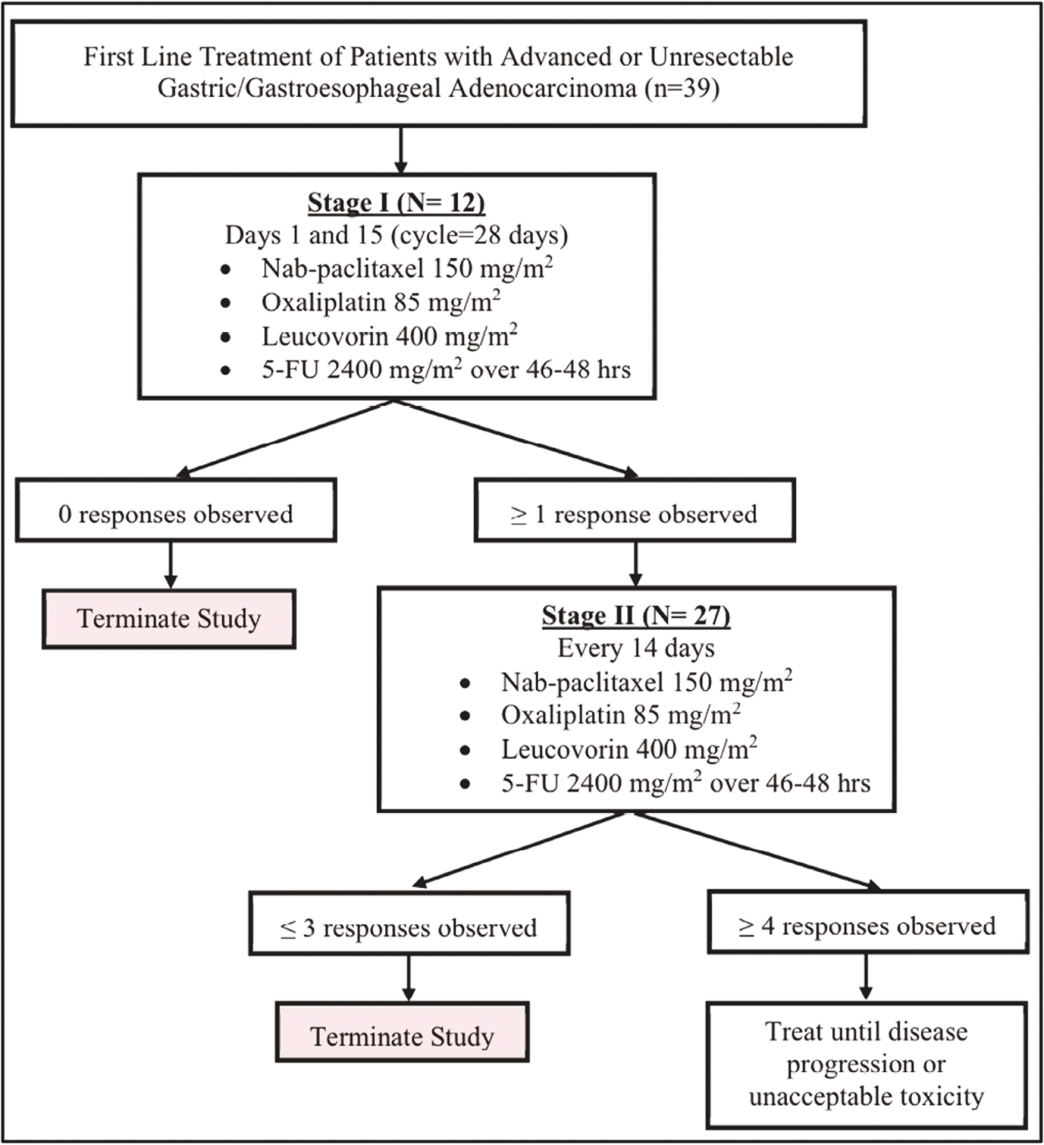
Study schema.

The study schema is shown in Figure 1. Stage 1 of the study completed the recruitment of 12 patients. When more than one patient achieved a response, recruitment continued to complete enrollment of 39 patients.

### Assessments and endpoints

Computed tomography or magnetic resonance imaging scans of the chest, abdomen, and pelvis were performed at screening and every odd-numbered cycle starting with cycle 3 until progressive disease (PD) or study withdrawal. Tumor assessment was performed by investigator assessment according to RECIST v1.1. Safety was assessed as the incidences of treatment-emergent adverse events and serious adverse events graded according to the National Cancer Institute’s Common Terminology Criteria for Adverse Events, (NCI CTCAE) version 4.

The primary endpoint was the objective response rate (ORR) calculated by combining the number of subjects who achieved complete response (CR) and partial response (PR) per RECIST 1.1 criteria. Secondary endpoints included median overall survival (OS), progression-free survival (PFS), time to progression (TTP), best ORR, and disease control rate, as well as grade 3 and 4 toxicities as defined by the NCI CTCAE version 4.

### Statistical analysis

This trial used Simon’s optimal 2-stage design to test the null hypothesis ORR = 5% versus the alternative ORR = 20% with 90% power and α = 0.10. If ≥1 responses were observed among *n* = 12 patients enrolled in stage 1, the trial would enroll an additional 25 patients for a total of *n* = 37, and the treatment would be considered promising, rejecting the null hypothesis, if ≥4 out of 37 responses were observed. ORR was estimated as the proportion of patients with CR/PR as their best response among patients evaluable for response and is reported with the corresponding Clopper-Pearson 95% CI. Descriptive statistics were used to summarize patient characteristics and treatment administration. TTP, PFS, and OS were estimated using the Kaplan-Meier method. Toxicity data were summarized for each AE type as the highest-grade toxicity experienced by the patient, and the number of patients experiencing each AE type is reported. Forest plots were used to summarize the objective response rate and median PFS and OS, along with the corresponding 95% CIs that were reported in other studies with similar regimens.

## Results

### Patients

Between 2017 and 2023, 39 patients with advanced/metastatic HER2-negative unresectable gastric/GEJ adenocarcinoma were enrolled in 6 BIG10 centers to receive FOLFOX-A. Baseline patient characteristics are summarized in Table 1. Twenty-one patients (57%) had gastric adenocarcinoma, 16 (43%) had GEJ adenocarcinoma, and 2 were listed as unknown.

**Table 1.** Patient characteristics.

Characteristic	*N* = 39^1^
Age	63 (20, 80)
Gender	
Female	9 (23%)
Male	30 (77%)
Race	
Asian	1 (2.8%)
Black or African American	1 (2.8%)
White	34 (94%)
Unknown	3
Ethnicity	
Hispanic or Latino	2 (5.6%)
Non-Hispanic	34 (94%)
Unknown	3
Tumor location	
Gastric	21 (57%)
GE junction	16 (43%)
Unknown	2
ECOG	
0	20 (51%)
1	19 (49%)
PD-L1 (%)	
≤1	8 (33%)
>1	16 (67%)
Unknown	15
T stage	
T3	13 (35%)
T4	5 (14%)
Tx	19 (51%)
Unknown	2
N stage	
N0	4 (11%)
N1	4 (11%)
N2	6 (16%)
N3	3 (8.1%)
NX	20 (54%)
Unknown	2
M stage	
M0	2 (5.4%)
M1	35 (95%)
Unknown	2

^1^Median (range); n (%).

Of the 39 patients enrolled, 38 started study therapy. The median number of cycles completed was 4.5 (IQR: 2.0-7.8), with 4 patients continuing study therapy for greater than 25 cycles. Of those who started treatment, 35 were evaluable for response, whereas 3 patients did not have a post-baseline response assessment (one died of multi-organ failure, one withdrew to pursue alternate therapy, and one withdrew for undisclosed reasons). Patients came off treatment due to disease progression (54%), alternative cancer therapy (18%), withdrawal after therapy start (10%), and adverse events/side effects/complications (7.7%).

### Efficacy

Objective radiographic response was achieved in 15 of 35 patients (ORR 42.9%) who were evaluable for response. One patient achieved CR (2.9%), 14 (40.0%) achieved PR, and 13 (37%) achieved SD. The median follow-up duration was 27 months (range: 18.2-51.9 months for censored patients). Although the number of patients evaluable for response fell short of the planned sample size *n* = 37, the number of responses exceeds the 4 responses that would be required to reject the null hypothesis, and the observed response rate exceeds the 20% ORR under the alternative hypothesis.

**Figure 2.**
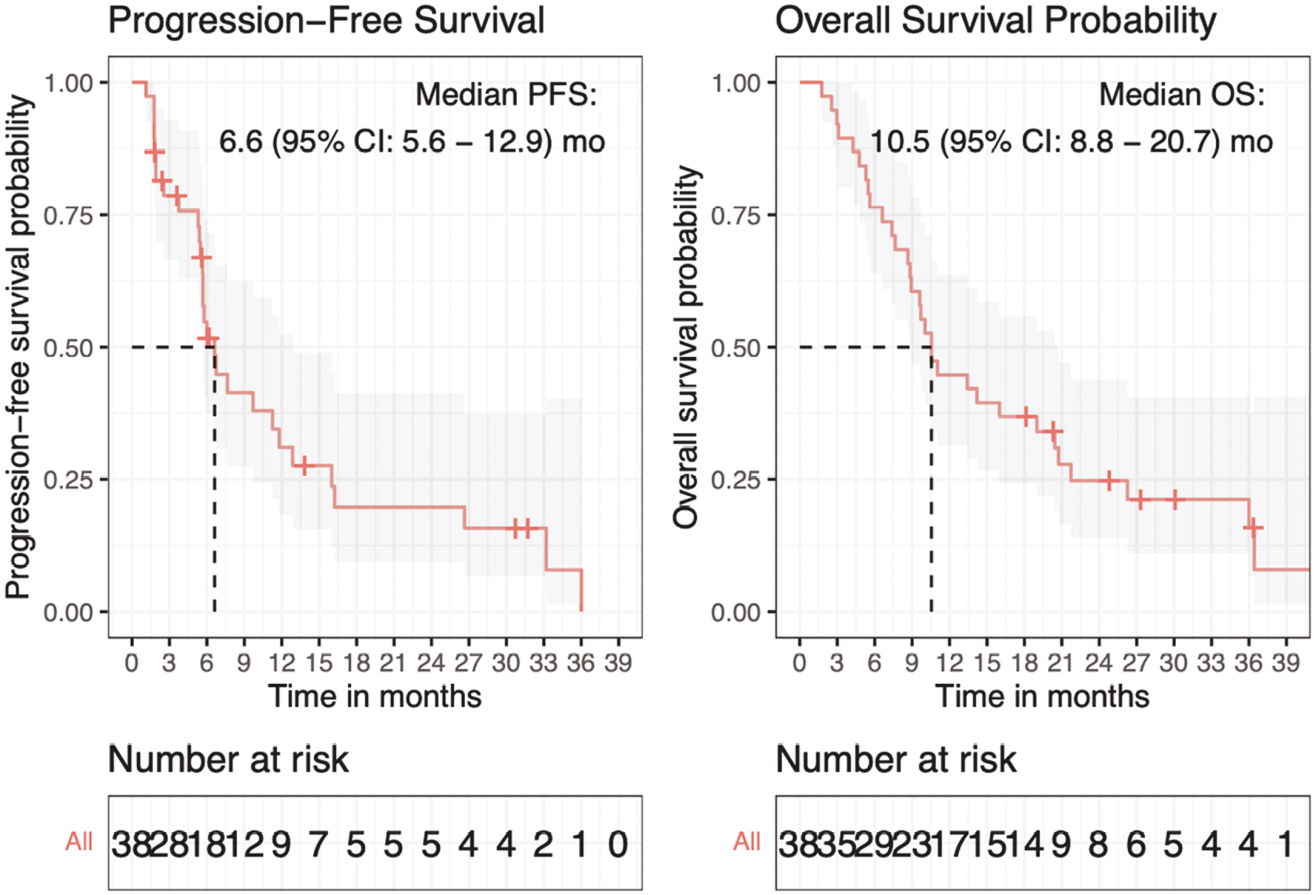
Kaplan-Meier curves for progression-free survival (PFS) and overall survival (OS).

**Figure 3.**
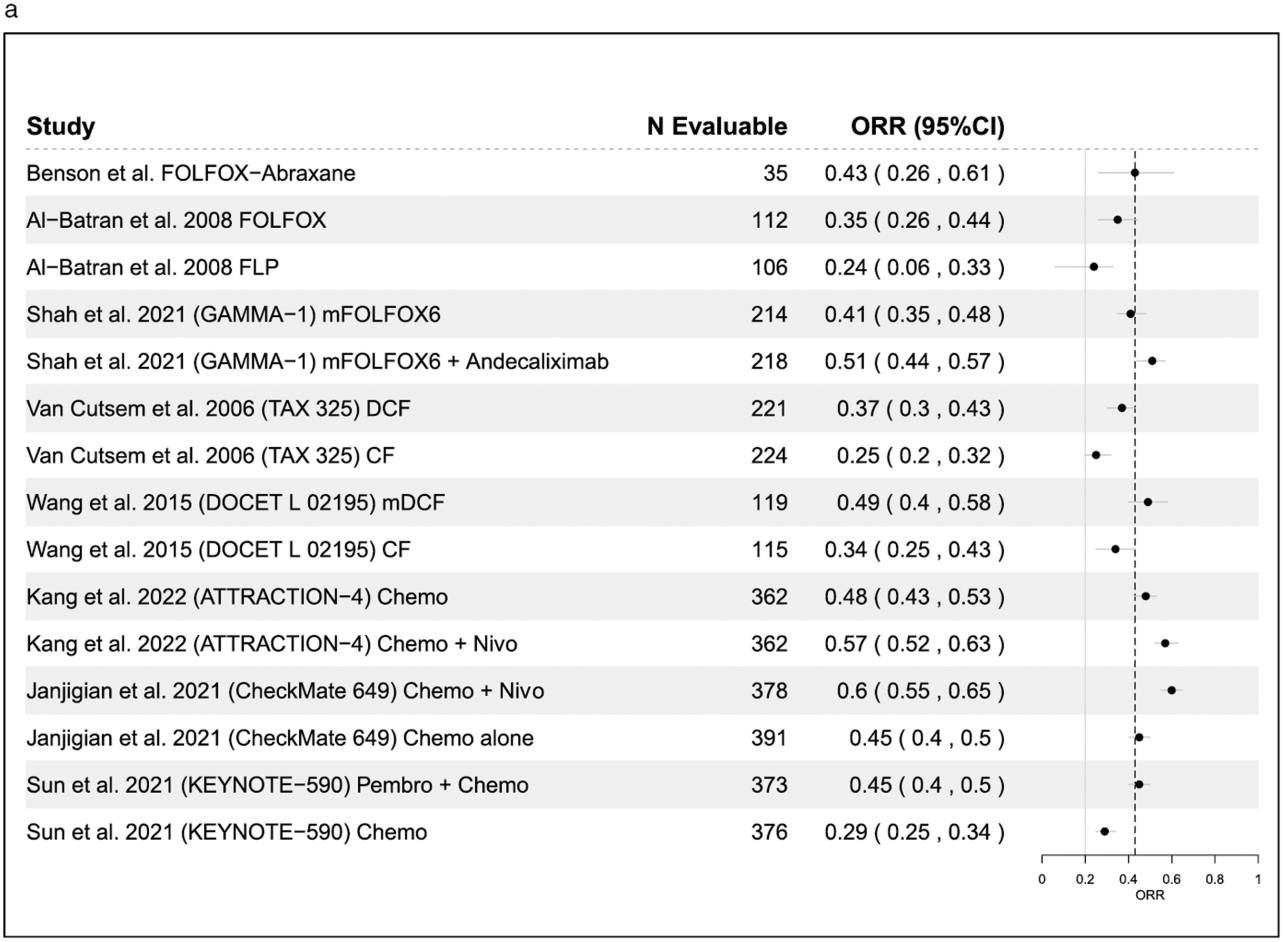

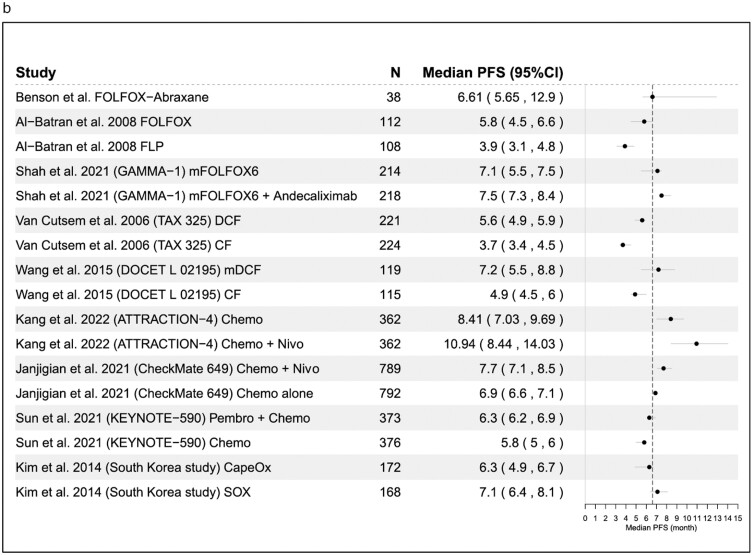

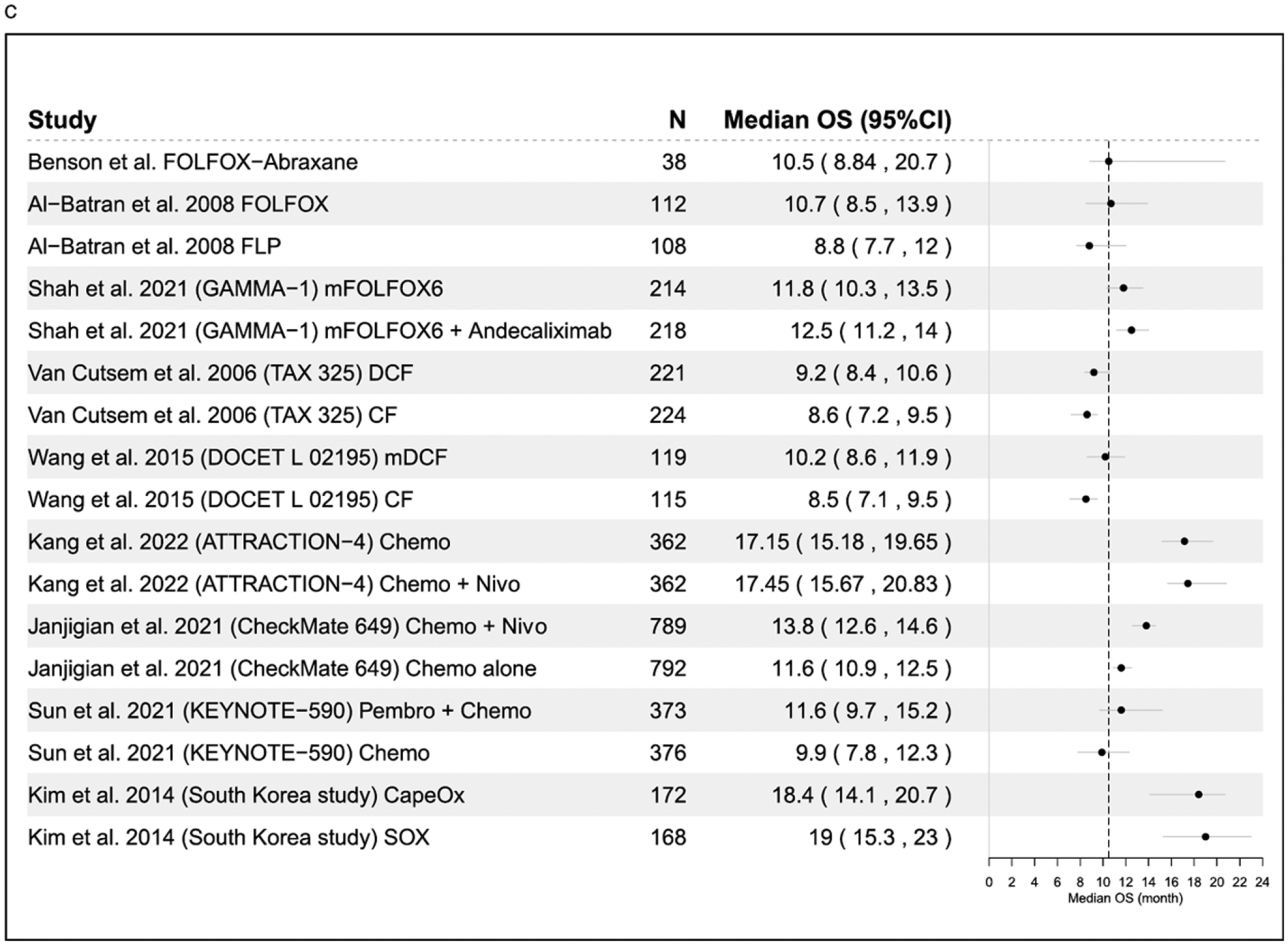
(a) Objective response rates (ORR) across similar studies. (b) Median progression-free survival (mPFS) across similar studies. (c) Median overall survival (mOS) across similar studies.

The median TTP was 11.3 months (95% CI: 5.7-26.7). The median PFS was 6.6 (95% CI: 5.6-12.9) months (Figure 2) and the median OS was 10.5 (95% CI: 8.9-21.7) months (Figure 3). PFS probability for FOLFOX-A at 6, 12, and 24 months was 51.7, 31.0, and 19.7 months, respectively. OS probability for FOLFOX-A at 6, 12, and 24 months was 76.3, 44.7, and 24.7 months, respectively. There were 31 deaths. There were no differences in ORR, PFS, and OS between gastric and GEJ patients (Supplementary Figure S1).

### Safety

Expected toxicities to FOLFOX and nab-paclitaxel of grade 1-2 peripheral neuropathy, diarrhea, and fatigue each occurred in about half of the study participants. The most common grade 3 or 4 adverse events were peripheral sensory neuropathy (18.4%), anemia (18.4%), neutropenia (15.8%), diarrhea (7.9%), and lymphopenia (7.9%). Table 2 demonstrates treatment-related adverse events for the study population. Treatment-related dose adjustments occurred in 25/38 (65.8%) of patients who started treatment. A complete list of treatment-related adverse events of any grade can be found in Supplementary Table S1; grade 3 or 4 adverse events can be found in Supplementary Table S2.

**Table 2.** Treatment-related adverse events.

AE term	Worst grade AE, related				
	1	2	3	4	Any grade
Peripheral sensory neuropathy	6 (15.8%)	14 (36.8%)	7 (18.4%)	0 (0%)	27 (71.1%)
Fatigue	12 (31.6%)	10 (26.3%)	2 (5.3%)	0 (0%)	24 (63.2%)
Diarrhea	15 (39.5%)	4 (10.5%)	2 (5.3%)	1 (2.6%)	22 (57.9%)
Nausea	11 (28.9%)	6 (15.8%)	2 (5.3%)	0 (0%)	19 (50.0%)
Anemia	6 (15.8%)	4 (10.5%)	7 (18.4%)	0 (0%)	17 (44.7%)
Vomiting	7 (18.4%)	7 (18.4%)	1 (2.6%)	0 (0%)	15 (39.5%)
Neutropenia	0 (0%)	5 (13.2%)	4 (10.5%)	2 (5.3%)	11 (28.9%)
Leukopenia	2 (5.3%)	5 (13.2%)	2 (5.3%)	0 (0%)	9 (23.7%)
Anorexia	6 (15.8%)	2 (5.3%)	1 (2.6%)	0 (0%)	9 (23.7%)
Lymphopenia	2 (5.3%)	2 (5.3%)	3 (7.9%)	0 (0%)	7 (18.4%)
Dehydration	2 (5.3%)	4 (10.5%)	1 (2.6%)	0 (0%)	7 (18.4%)
Hypokalemia	2 (5.3%)	2 (5.3%)	1 (2.6%)	0 (0%)	5 (13.2%)

## Discussion

In this phase II trial, we evaluated the clinical outcomes and safety profile of a modified triplet chemotherapy regimen with FOLFOX-A in patients with metastatic or advanced unresectable HER2 negative gastric/GEJ adenocarcinoma.

FOLFOX-A demonstrated similar clinical outcomes to other doublet and triplet chemotherapy regimens that have been studied in this patient population (Figure 3a-c).^5,6,8,9,16-19^

Perhaps most notable were the hematologic side effects for this triplet regimen when compared to historical controls for other combination cytotoxic chemotherapy regimens. In the FOLFOX-A regimen the risk of grade 3 or 4 leukopenia (5.3%, as defined by white cell count < 2.0 × 10^9^/L) and neutropenia (15.8%, as defined by absolute neutrophil count < 1.0 × 10^9^/L) was comparable to that in the 5-FU/leucovorin and oxaliplatin regimen (FLO), 6.3% and 11.6% respectively,^5^ and more favorable than in the 5-FU/leucovorin, oxaliplatin, and docetaxel (FLOT) regimen, 27% and 51%, respectively.^20^ While not an aim of this trial, comparisons to additional hematologic side-effect profiles from previous doublet and triplet regimens are included in Supplementary Table S3.

Since the initiation of this trial, there have been major advances in the treatment of metastatic HER2-negative gastric/GEJ adenocarcinoma with the addition of immune checkpoint inhibitors to chemotherapy in the frontline setting. Nivolumab has FDA approval in this setting based on CheckMate 649, a phase III trial that evaluated nivolumab and chemotherapy versus chemotherapy alone. The addition of nivolumab to chemotherapy demonstrated a median OS of 13.8 months (95% CI: 12.6-14.6) versus 11.6 months (95% CI: 10.9-12.5) in the chemotherapy alone arm (HR 0.80; 95% CI: 0.71-0.90) regardless of PD-L1 combined positive score.^18^ Objective response rate was 58% (350/603) in nivolumab group compared to 46% (280/608) in chemotherapy alone group. Additionally, pembrolizumab is approved in combination with chemotherapy for patients with metastatic or locally advanced GEJ adenocarcinoma based on KEYNOTE-590 showing improved OS in the pembrolizumab plus chemotherapy group than in the chemotherapy plus placebo group (11.6 months, 95% CI: 9.7-15.2 vs 9.9 months, 95% CI: 7.8-12.3; HR 0.74, 95% CI: 0.54-1.02).^19^ Objective response rate was 45% (168/373) in pembrolizumab and chemotherapy group compared to 29.3% (110/376) in chemotherapy plus placebo group. While these advancements are important, some patients will not be candidates for immunotherapy for a variety of reasons including autoimmune history, preexisting need for immunosuppression, or immune-related adverse events. With a median overall survival of less than 1 year across current doublet and triplet chemotherapy regimens, more options are needed for patients who are unable to tolerate immunotherapy.

## Conclusion

In summary, the addition of FOLFOX-A resulted in an overall objective response of 42.9% which exceeded the expected study target of 20%, had expected toxicities, and extended disease control for some patients. The favorable hematologic toxicity profile of FOLFOX-A as compared to FLOT is worthy of further study of FOLFOX-A as a first-line therapy for metastatic HER2-negative gastric/GEJ adenocarcinoma. Future investigations could consider incorporating FOLFOX-A chemotherapy backbone in combination with immunotherapy.

## Supplementary material

Supplementary material is available at *The Oncologist* online.

oyae236_suppl_Supplementary_Figure

oyae236_suppl_Supplementary_Tables

## Data Availability

The data underlying this article will be shared on reasonable request to the corresponding author.
