# Supplementary material for: A phase II study of FOLFOX combined with nab-paclitaxel in the treatment of metastatic or advanced unresectable gastric, gastroesophageal junction adenocarcinoma: a Big Ten Cancer Research Consortium trial
Source: Oncologist. 2024 Sep 18;29(12):1044–50. doi: 10.1093/oncolo/oyae236 (PMC11630795; doi:10.1093/oncolo/oyae236)
Supplement: oyae236_suppl_Supplementary_Figure [file oyae236_suppl_supplementary_figure.docx]

**Supplemental Figure 1.** Kaplan Meier Curves for Progression Free Survival (PFS) and Overall Survival (OS), By Histology

**
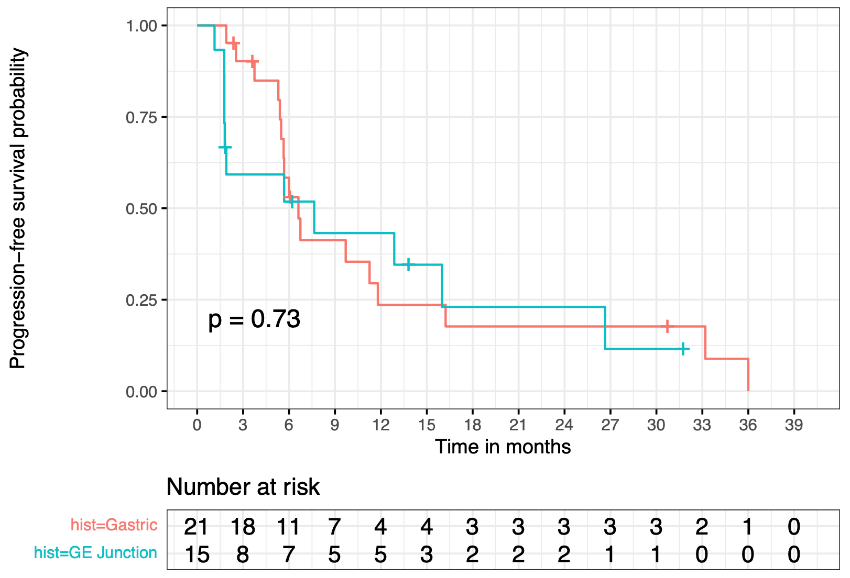

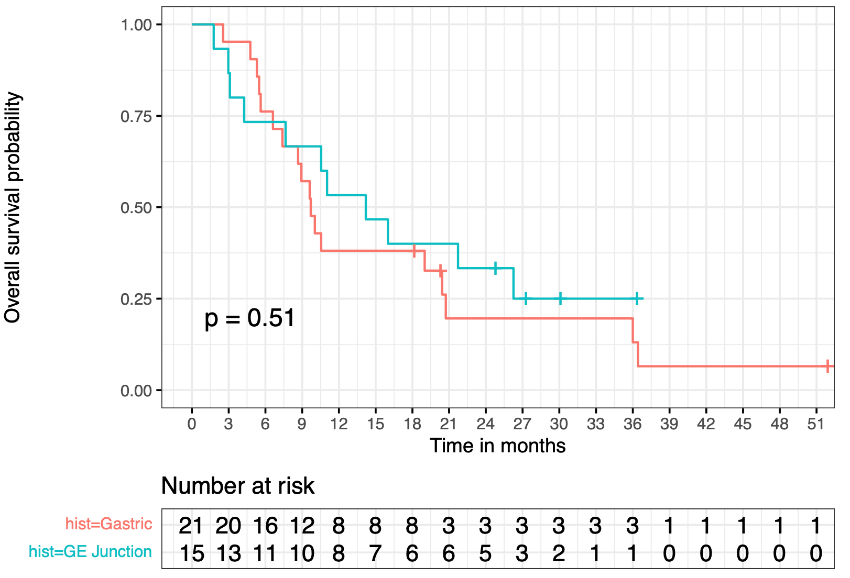
**
