# Supplementary material for: A phase II study of FOLFOX combined with nab-paclitaxel in the treatment of metastatic or advanced unresectable gastric, gastroesophageal junction adenocarcinoma: a Big Ten Cancer Research Consortium trial
Source: Oncologist. 2024 Sep 18;29(12):1044–50. doi: 10.1093/oncolo/oyae236 (PMC11630795; doi:10.1093/oncolo/oyae236)
Supplement: oyae236_suppl_Supplementary_Tables [file oyae236_suppl_supplementary_tables.docx]

## **SUPPLEMENTAL Table 1. Treatment-related Adverse Events (Any Grade)**

|  | Worst Grade AE, Related | | |
| --- | --- | --- | --- |
| AE Term | 1/2 | 3/4 | Any Grade |
| Peripheral sensory neuropathy | 20 (52.6%) | 7 (18.4%) | 27 (71.1%) |
| Fatigue | 22 (57.9%) | 2 (5.3%) | 24 (63.2%) |
| Diarrhea | 19 (50.0%) | 3 (7.9%) | 22 (57.9%) |
| Nausea | 17 (44.7%) | 2 (5.3%) | 19 (50.0%) |
| Anemia | 10 (26.3%) | 7 (18.4%) | 17 (44.7%) |
| Vomiting | 14 (36.8%) | 1 (2.6%) | 15 (39.5%) |
| Alopecia | 15 (39.5%) | 0 | 15 (39.5%) |
| Neutropenia | 5 (13.2%) | 6 (15.8%) | 11 (28.9%) |
| Constipation | 10 (26.3%) | 0 | 10 (26.3%) |
| Mucositis oral | 10 (26.3%) | 0 | 10 (26.3%) |
| Leukopenia | 7 (18.4%) | 2 (5.3%) | 9 (23.7%) |
| Anorexia | 8 (21.1%) | 1 (2.6%) | 9 (23.7%) |
| Lymphopenia | 4 (10.5%) | 3 (7.9%) | 7 (18.4%) |
| Dehydration | 6 (15.8%) | 1 (2.6%) | 7 (18.4%) |
| Dysgeusia | 7 (18.4%) | 0 | 7 (18.4%) |
| Platelet count decreased | 7 (18.4%) | 0 | 7 (18.4%) |
| Hypokalemia | 4 (10.5%) | 1 (2.6%) | 5 (13.2%) |
| Dyspepsia | 5 (13.2%) | 0 | 5 (13.2%) |
| Edema limbs | 5 (13.2%) | 0 | 5 (13.2%) |
| Weight loss | 5 (13.2%) | 0 | 5 (13.2%) |
| Alanine aminotransferase increased | 4 (10.5%) | 0 | 4 (10.5%) |
| Aspartate aminotransferase increased | 4 (10.5%) | 0 | 4 (10.5%) |
| Bloating | 4 (10.5%) | 0 | 4 (10.5%) |
| Dizziness | 4 (10.5%) | 0 | 4 (10.5%) |
| Hypomagnesemia | 4 (10.5%) | 0 | 4 (10.5%) |
| Myalgia | 4 (10.5%) | 0 | 4 (10.5%) |
| Hypophosphatemia | 2 (5.3%) | 1 (2.6%) | 3 (7.9%) |
| Abdominal pain | 3 (7.9%) | 0 | 3 (7.9%) |
| Alkaline phosphatase increased | 3 (7.9%) | 0 | 3 (7.9%) |
| Infections and infestations - other, specify | 3 (7.9%) | 0 | 3 (7.9%) |
| Infusion related reaction | 3 (7.9%) | 0 | 3 (7.9%) |
| Pain in extremity | 3 (7.9%) | 0 | 3 (7.9%) |
| Blood and lymphatic system disorders - other, specify | 1 (2.6%) | 1 (2.6%) | 2 (5.3%) |
| Arthralgia | 2 (5.3%) | 0 | 2 (5.3%) |
| Bone pain | 2 (5.3%) | 0 | 2 (5.3%) |
| Dry skin | 2 (5.3%) | 0 | 2 (5.3%) |
| Epistaxis | 2 (5.3%) | 0 | 2 (5.3%) |
| Gastroesophageal reflux disease | 2 (5.3%) | 0 | 2 (5.3%) |
| General disorders and administration site conditions - other, specify | 2 (5.3%) | 0 | 2 (5.3%) |
| Generalized muscle weakness | 2 (5.3%) | 0 | 2 (5.3%) |
| Headache | 2 (5.3%) | 0 | 2 (5.3%) |
| Hearing impaired | 2 (5.3%) | 0 | 2 (5.3%) |
| Hematuria | 2 (5.3%) | 0 | 2 (5.3%) |
| Hiccups | 2 (5.3%) | 0 | 2 (5.3%) |
| Hypoalbuminemia | 2 (5.3%) | 0 | 2 (5.3%) |
| Memory impairment | 2 (5.3%) | 0 | 2 (5.3%) |
| Muscle weakness lower limb | 2 (5.3%) | 0 | 2 (5.3%) |
| Musculoskeletal and connective tissue disorder - other, specify | 2 (5.3%) | 0 | 2 (5.3%) |
| Nervous system disorders - other, specify | 2 (5.3%) | 0 | 2 (5.3%) |
| Palmar-plantar erythrodysesthesia syndrome | 2 (5.3%) | 0 | 2 (5.3%) |
| Peripheral motor neuropathy | 2 (5.3%) | 0 | 2 (5.3%) |
| Rash acneiform | 2 (5.3%) | 0 | 2 (5.3%) |
| Rash maculo-papular | 2 (5.3%) | 0 | 2 (5.3%) |
| Colitis | 0 | 1 (2.6%) | 1 (2.6%) |
| Depression | 0 | 1 (2.6%) | 1 (2.6%) |
| Lung infection | 0 | 1 (2.6%) | 1 (2.6%) |
| Paresthesia | 0 | 1 (2.6%) | 1 (2.6%) |
| Pericardial tamponade | 0 | 1 (2.6%) | 1 (2.6%) |
| Pericarditis | 0 | 1 (2.6%) | 1 (2.6%) |
| Skin infection | 0 | 1 (2.6%) | 1 (2.6%) |
| Allergic reaction | 1 (2.6%) | 0 | 1 (2.6%) |
| Back pain | 1 (2.6%) | 0 | 1 (2.6%) |
| Blurred vision | 1 (2.6%) | 0 | 1 (2.6%) |
| Catheter related infection | 1 (2.6%) | 0 | 1 (2.6%) |
| Concentration impairment | 1 (2.6%) | 0 | 1 (2.6%) |
| Cough | 1 (2.6%) | 0 | 1 (2.6%) |
| Dry mouth | 1 (2.6%) | 0 | 1 (2.6%) |
| Dyspnea | 1 (2.6%) | 0 | 1 (2.6%) |
| Ear and labyrinth disorders - other, specify | 1 (2.6%) | 0 | 1 (2.6%) |
| Erythroderma | 1 (2.6%) | 0 | 1 (2.6%) |
| Eye disorders - other, specify | 1 (2.6%) | 0 | 1 (2.6%) |
| Fall | 1 (2.6%) | 0 | 1 (2.6%) |
| Flu like symptoms | 1 (2.6%) | 0 | 1 (2.6%) |
| Gastric ulcer | 1 (2.6%) | 0 | 1 (2.6%) |
| Gingival pain | 1 (2.6%) | 0 | 1 (2.6%) |
| Hoarseness | 1 (2.6%) | 0 | 1 (2.6%) |
| Hyperglycemia | 1 (2.6%) | 0 | 1 (2.6%) |
| Hypermagnesemia | 1 (2.6%) | 0 | 1 (2.6%) |
| Hyperuricemia | 1 (2.6%) | 0 | 1 (2.6%) |
| Hyponatremia | 1 (2.6%) | 0 | 1 (2.6%) |
| Hypotension | 1 (2.6%) | 0 | 1 (2.6%) |
| Investigations - other, specify | 1 (2.6%) | 0 | 1 (2.6%) |
| Localized edema | 1 (2.6%) | 0 | 1 (2.6%) |
| Lower gastrointestinal hemorrhage | 1 (2.6%) | 0 | 1 (2.6%) |
| Nail discoloration | 1 (2.6%) | 0 | 1 (2.6%) |
| Nail ridging | 1 (2.6%) | 0 | 1 (2.6%) |
| Oral dysesthesia | 1 (2.6%) | 0 | 1 (2.6%) |
| Oral pain | 1 (2.6%) | 0 | 1 (2.6%) |
| Pain | 1 (2.6%) | 0 | 1 (2.6%) |
| Periorbital edema | 1 (2.6%) | 0 | 1 (2.6%) |
| Pneumonitis | 1 (2.6%) | 0 | 1 (2.6%) |
| Postnasal drip | 1 (2.6%) | 0 | 1 (2.6%) |
| Skin and subcutaneous tissue disorders - other, specify | 1 (2.6%) | 0 | 1 (2.6%) |
| Skin hyperpigmentation | 1 (2.6%) | 0 | 1 (2.6%) |
| Stomach pain | 1 (2.6%) | 0 | 1 (2.6%) |
| Syncope | 1 (2.6%) | 0 | 1 (2.6%) |
| Tinnitus | 1 (2.6%) | 0 | 1 (2.6%) |
| Upper respiratory infection | 1 (2.6%) | 0 | 1 (2.6%) |
| Weight gain | 1 (2.6%) | 0 | 1 (2.6%) |
| Wound infection | 1 (2.6%) | 0 | 1 (2.6%) |

## **SUPPLEMENTAL Table 2. Treatment-related Adverse Events (Grade 3/4)**

|  | Worst Grade AE, Related |
| --- | --- |
| AE Term | 3/4 |
| Peripheral sensory neuropathy | 7 (18.4%) |
| Fatigue | 2 (5.3%) |
| Diarrhea | 3 (7.9%) |
| Nausea | 2 (5.3%) |
| Anemia | 7 (18.4%) |
| Vomiting | 1 (2.6%) |
| Neutropenia | 6 (15.8%) |
| Leukopenia | 2 (5.3%) |
| Anorexia | 1 (2.6%) |
| Lymphopenia | 3 (7.9%) |
| Dehydration | 1 (2.6%) |
| Hypokalemia | 1 (2.6%) |
| Hypophosphatemia | 1 (2.6%) |
| Blood and lymphatic system disorders - other, specify | 1 (2.6%) |
| Colitis | 1 (2.6%) |
| Depression | 1 (2.6%) |
| Lung infection | 1 (2.6%) |
| Paresthesia | 1 (2.6%) |
| Pericardial tamponade | 1 (2.6%) |
| Pericarditis | 1 (2.6%) |
| Skin infection | 1 (2.6%) |

**SUPPLEMENTAL Table 3. Side effect profiles for similar doublet and triplet regimens**

| **Regimen** | **Study** | **Years** | **Phase** | **Comparator** | **HEME Side Effects (G3, higher)** | **OTHER Side Effects (G3, higher)** | **Regimen for SE** |
| --- | --- | --- | --- | --- | --- | --- | --- |
| FOLFOX-Abraxane | [Our study](https://ascopubs.org/doi/10.1200/JCO.2022.40.4_suppl.283) | 2017-2023 | Phase 2 | N/A | Anemia (18.4%) Leukopenia (5.3%) Neutropenia (15.8%) | PN (18.4%) Diarrhea (7.9%) Fatigue (5.3%) Nausea (5.3%) | FOLFOX |
| FOLFOX (FLO) | [Al-Batran et al. 2008](https://doi.org/10.1200/jco.2007.13.9378) | 2003-2006 | Phase 3 | FLP (Fluorouracil, Leucovorin, Cispltain) | Anemia (2.7%) Leukopenia (6.3%) Neutropenia (11.6%) | PN (14.3%) Diarrhea (6.3%) Fatigue (3.6%) Nausea (4.5%) | FOLFOX (FLO) |
| FLOT | [Al-Batran et al, 2018](https://www.thelancet.com/journals/lancet/article/PIIS0140-6736(18)32557-1/fulltext) | 2010-2015 | Phase 3 | ECF/ECX | Anemia (3%) Leukopenia (27%) Neutropenia (51%) | PN (7%) Diarrhea (10%) Fatigue (NR) Nausea (7%) | FLOT |
| mFOLFOX6 & Andecaliximab | [Shah et al. 2021 (GAMMA-1)](https://www.ncbi.nlm.nih.gov/pmc/articles/pmc8078292/) | 2015-2019 | Phase 3 (C) | mFOLFOX6 | Anemia (11%) Leukopenia (12%) Neutropenia (29%) | PN (2%) Diarrhea (4%) Fatigue (9%) Nausea (3%) | FOLFOX |
| Nivo + CapeOx/SOX | [Kang et al. 2022 (ATTRACTION-4)](https://doi.org/10.1016/s1470-2045(21)00692-6) | 2017-2018 | Phase 3 | CapeOx/SOX | Anemia (18%) Leukopenia (3%) Neutropenia (3%) | PN (2%) Diarrhea (4%) Fatigue (<1%%) Nausea (3%) | SOX/Capeox |
| mFOLFOX6/CapeOx + Nivo | [Janjigian et al. 2021 (CheckMate 649)](https://www.ncbi.nlm.nih.gov/pmc/articles/pmc8436782/) | 2017-2019 | Phase 3 | CapeOx/FOLFOX | Anemia (3%) Leukopenia (2%) Neutropenia (12%) | PN (3%) Diarrhea (3%) Fatigue (2%%) Nausea (2%) | mFOLFOX6 or CapeOx |
| DCF | [Van Cutsem et al. 2006 (TAX 325)](https://doi.org/10.1200/jco.2006.06.8429) | 1999-2003 | Phase 3 | CF | Anemia (18%) Leukopenia (65%) Neutropenia (82%) | PN (8%) Diarrhea (19%) Fatigue (19%) Nausea (14%) | DCF |
| mDCF | [Wang et al. 2015 (DOCET L 02195)](https://www.ncbi.nlm.nih.gov/pmc/articles/PMC4688303/) | 2008-2010 | Phase 3 | CF | Anemia (5.0%) Leukopenia (52.1%) Neutropenia (60.5%) | PN (NR) Diarrhea (12.6%) Fatigue (NR) Nausea (2.5%) | mDCF |
| 5FU/cis + Pembro | [Sun et al. 2021 (KEYNOTE-590)](https://pubmed.ncbi.nlm.nih.gov/34454674/) | 2017-2019 | Phase 3 | 5FU/Cis | Anemia (15%) Leukopenia (5%) Neutropenia (17%) | PN (0%) Diarrhea (2%) Fatigue (5%) Nausea (6%) | 5FU/cis |
